# Clinical features and genotype-phenotype correlation analysis in patients with *ATL1* mutations: A literature reanalysis

**DOI:** 10.1186/s40035-017-0079-3

**Published:** 2017-04-04

**Authors:** Guo-hua Zhao, Xiao-min Liu

**Affiliations:** 1 Department of Neurology, Second Affiliated Hospital, School of Medicine, Zhejiang University, Hangzhou, 310009 China; 2 Department of Neurology, Fourth Affiliated Hospital, School of Medicine, Zhejiang University, Yiwu, 322000 China; 3 Department of Neurology, Qianfoshan Hospital, Shandong University, Jinan, 16766 China

**Keywords:** Hereditary spastic paraplegia, SPG3A, Age at onset, *ATL1*, Mutation, Genotype-phenotype correlation

## Abstract

**Background:**

The hereditary spastic paraplegias (HSPs) are a group of clinically and genetically heterogeneous disorders. Approximately 10% of the autosomal dominant (AD) HSPs (ADHSPs) have the spastic paraplegia 3A (SPG3A) genotype which is caused by *ATL1* gene mutations. Currently there are more than 60 reported *ATL1* gene mutations and the genotype-phenotype correlation remains unclear. The study aims to investigate the genotype-phenotype correlation in SPG3A patients.

**Methods:**

We performed a reanalysis of the clinical features and genotype-phenotype correlations in 51 reported studies exhibiting an *ATL1* gene mutation.

**Results:**

Most HSPs-SPG3A patients exhibited an early age at onset (AAO) of <10 years old, and showed an autosomal dominant pure spastic paraplegia. We found that 14% of the HSPs-SPG3A patients presented complicated phenotypes, with distal atrophy being the most common complicated symptom. The AAO of each mutation group was not statistically significant (*P* > 0.05). The mutational spectrum associated with *ATL1* gene mutation is wide, and most mutations are missense mutations, but do not involve the functional motif of *ATL1* gene encoded atlastin-1 protein.

**Conclusions:**

Our findings indicate that there is no clear genotype-phenotype correlation in HSPs-SPG3A patients. We also find that exons 4, 7, 8 and 12 are mutation hotspots in *ATL1* gene.

**Electronic supplementary material:**

The online version of this article (doi:10.1186/s40035-017-0079-3) contains supplementary material, which is available to authorized users.

## Background

The hereditary spastic paraplegias (HSPs) are a group of clinically heterogeneous neurological disorders, which are classified into “pure” or “complicated” HSP according to the clinical features. The pure HSP is defined by progressive spasticity and weakness limited to the lower limbs, while the complicated HSP may include other neurological manifestations such as optic atrophy, retinal pigmentation, seizures, deafness, neuropathy and mental retardation. In the clinic, HSPs can also be classified into early onset (mainly 1^st^ decade of life) and late onset (between the 2^nd^ and 4^th^ decade) type. The main pathological changes of HSP include the axonal degeneration of the corticospinal tracts and back column [1, 2].

Genetic mutations are the main cause of HSPs and there are currently over 72 spastic paraplegia genes or genetic loci (designated SPG1-SPG72 genetic type in order of their discovery) in which mutations can occur [3]. HSPs can be inherited as autosomal dominant (AD), autosomal recessive (AR) or X-linked trait or a spastic paraplegia syndrome. Among identified mutations, approximately 40% of definite autosomal dominant pure HSP mutations are in the spastic paraplegia 4 (*SPG4/SPAST*) gene which encodes the spastin protein [4, 5].

SPG3A is the second most common type of HSP which accounts for approximately 10% of autosomal dominant HSP [6] and is caused by mutations in the atlastin-1 (*ATL1*) gene. The atlastin-1 protein is a member of the dynamin family of large guanosine triphosphatases (GTPases) which contains three conserved motif-P loops (74GAFRKGKS81), RD (217RD) and DxxG (146DTQG) which are characteristic regions for guanylate binding/GTPase active sites [6]. HSPs-SPG3A phenotype (HSPs with *ATL1* gene mutations) was generally a pure HSP with age at onset (AAO) less than 10 years old [6]. Patients characterize progressive bilateral and mostly symmetric lower extremity weakness and spasticity.

Currently there are more than 60 different *ATL1* gene mutations described, including numerous missense, small deletion, small insertion and splice site mutations, as well as whole exon deletions [6–56]. However, the genotype-phenotype correlation remains unclear [13]. In this study, we perform a reanalysis of all published studies (*n* = 51) to identify the clinical features and then genotype-phenotype correlations in HSPs caused by *ATL1* gene mutations.

## Methods

We conducted a literature search using databases from PubMed (http://ncbi.nlm.nih.gov/pubmed) and the China National Knowledge Infrastructure (CNKI) (http://cnki.net) with the keyword “SPG3A” or “*ATL1*”, which resulted in 51 articles describing *ATL1* gene mutations [6–56]. We collected information related to the age at onset (AAO), age at examination, pure or complicated form, involvement of upper and lower limbs, Babinski signs, urinary urgency and other symptoms or signs for individually affected patients directly from relevant papers. Asymptomatic individuals were also included, but excluded from the analysis of AAO and pure or complicated form. Patients with elderly sensory neuropathy caused by *ATL1* gene mutations were excluded in this study [57, 58]. We reanalysed the clinical and genetic data in *ATL1* gene mutant patients and performed a correlation analysis of AAO with mutational class in *ATL1* gene. Comparisons of data were performed using two-way ANOVA. Tests were considered statistically significant for *P* < 0.05.

## Results

The patients’ clinical information and the *ATL1* gene mutation of 51 reports are summarized in Additional file 1: Table S1. The published studies contain data for 142 families with known *ATL1* gene mutations. These 142 families included 130 (91.54%) autosomal dominant HSP (ADHSP) families, 10 (7.04%) sporadic families, one (0.70%) ARHSP family, and one (0.70%) family with unknown inheritance mode. Gender information was available in 151 patients, including 88 male patients and 63 female patients (ratio is 1.40:1). The main clinical features included lower spasticity (99.68%, 313/314), upper spasticity (10.03%, 30/299), Babinski sign (87.83%, 231/263) and urinary urgency (16.37%, 38/232). AAO data were available in 355 subjects from infancy to the seventh decade, in which 301 (84.79%) patients had AAO < 10 years old, whereas 54 (15.21%) patients had AAO >10 years old. Patient information for pure or complicated type was available in 440 patients, including 378 (85.90%) pure HSP and 62 (14.10%) complicated HSP patients. Distal atrophy or neuropathy is the most common symptom in patients with complicated *SPG3A* gene mutations (69.35%, 43/62). In addition, 15/142 families (10.56%) showed incomplete penetrance.

**Fig. 1:**
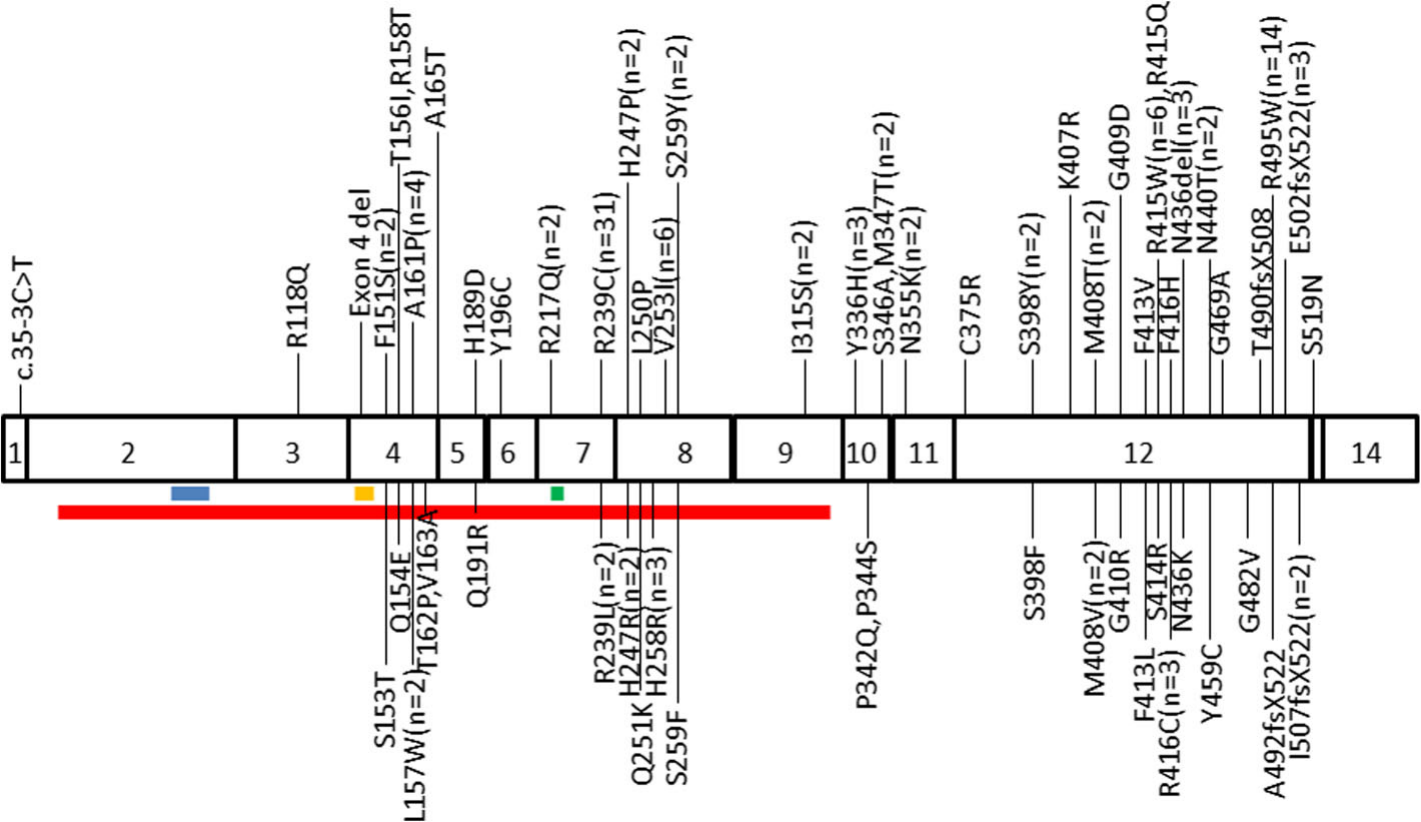
The *ATL1* mutation spectrum in CDS. *Red line* indicates the GBp/Ras-like GTPase domain; *blue line* shows the P-loop domain; *orange line* displays the DxxG domain; *green line* indicates the RD domain. n correlates with the number (if ≥2) of families containing *ATL1* mutations

In total, there were 61 different types of mutations reported, which were divided into five broad groups: 130 (91.54%) families had missense mutations (54 types), six (4.23%) had small insertions (4 types), four had (2.82%) small deletions (2 types), one (0.70%) had presumed splice site mutation (1 type), and one (0.70%) had whole exon deletion (1 type). The mutations were located in exon 3 (0.70%, *n* = 1), exon 4 (11.26%, *n* = 16), exon 5 (1.41%, *n* = 2), exon 6 (0.70%, *n* = 1), exon 7 (24.65%, *n* = 35), exon 8 (12.68%, *n* = 18), exon 9 (1.41%, *n* = 2), exon 10 (5.63%, *n* = 8), exon 11 (1.41%, *n* = 2), exon 12 (38.73%, *n* = 55), exon 13 (0.70%, *n* = 1), and intron 1 (0.70%, *n* = 1). A total of 124 (87.32%) mutations were found in exons 4, 7, 8 and 12. No mutations were detected in exons 1, 2 and 14. Figure 1 shows the locations of *ATL1* gene mutations and the number of mutations found in the families. Most mutations did not involve in the functional motif of atlastin-1, except R217Q, c.35-3C > T and exon 4 deletion [7, 13, 40]. The most commonly reported mutations were R239C (*n* = 31) and R495W (*n* =14). All the published mutations are listed in Ensemble database (ensemble.org).

The AAOs of each mutation group are summarized in Table 1. The patients with missense mutations had a slightly lower AAO, however this difference is not statistically significant (*F* = 1.273, *P* = 0.282). The patients with splice site mutation and exon deletion were not included in the statistics because there was only one patient in each group.

**Table 1:** The comparison of AAOs between different kinds of mutations

Mutation type	No. of families	No. of subjects	Mean age at onset (years)
Missense	109	271	7.47 ± 11.97
Small insertion	6	10	13.60 ± 12.39
Small deletion	4	12	8.25 ± 11.87
Splice site	1	1	44
Exon deletion	1	1	Not available

*F* = 1.273, *P* = 0.282. No.: number

## Discussion

HSPs-SPG3A patients account for approximately 10% of ADHSP. Although several large cohorts of patients with mutant *ATL1* gene were reported [17, 21, 27, 29, 32, 36, 45, 50, 54, 56], a genotype-phenotype correlation still remains unclear. Here, we reanalysed the observations on 142 families and confirmed three previously reported observations. First, we find that most HSPs-SPG3A patients exhibiting early AAO and autosomal dominant pure spastic paraplegia there have a wide mutational spectrum associated with *ATL1* gene mutations. Second, we find that most mutations are missense but do not involve the functional motifs of atlastin-1. Third, we note that exon 4, 7, 8 and 12 might be mutation hotspots. Additionally, we found that the complicated type was prevalent in HSPs-SPG3A patients, and that distal atrophy or neuropathy is the most common complicated symptoms.

*ATL1* gene mutations are thought to be the most common cause of hereditary spastic paraplegia with an AAO < 10 years [17]. Our re-analysis of the 51 reported studies showed that 84.79% patients exhibited early AAO (<10 years), but we found that 15.21% patients had a later AAO (>10 years). Therefore, *ATL1* gene mutation analysis should not be limited to early onset HSP [11, 34].

Our re-analysis also showed no correlation between AAO and mutational classes. More studies with larger sample sizes may be required to resolve this issue because of the limited number of small insertion, small deletion, splice site mutation and exon deletion. In addition, there was variability of AAOs between families with the same mutation, even within the same family. Some members in different families exhibiting the same mutation had different AAOs. For example, the AAOs for the A161P *ATL1* gene mutation could be childhood or age 45–55 in different families [11]. We found that both childhood-onset HSP and late onset HSP (after age 40 years) occurred in the same family with A161P mutation [21]. Furthermore, intrafamilial variability in AAO varied from eight to 28 years in a family with R495W mutation [21]. One family member with complicated HSP showed AAOs >30 years, whereas another family member with pure HSP presented AAO at puberty, but both members had a R416C mutation in the *ATL1* gene, suggesting a clear intrafamilial variability. Hedera et al. reported a family in which patients had a variable AAO from five to 39 years, and two subjects were functionally asymptomatic despite abnormalities in neurological examinations [15]. Patients in an ADHSP family carrying the *ATL1* R416C mutation were found to have variable clinical characteristics, both the pure phenotype with early onset and the complicated phenotype with later onset [41]. Differences of AAO and clinical features between families with the same mutation or in the same family might be due to variability in expression of this mutation or maybe to related to other genetic or epigenetic factors [11, 18, 34]. Overall, the comparison of the clinical data for all *ATL1* gene mutation families failed to reveal any genotype-phenotype correlation as demonstrated in other types of ADHSP [13].

*ATL1* gene was commonly thought to be associated with pure spastic paraplegia manifesting as lower limb spasticity, decreased vibration sense in the lower limbs, and sphincter disturbances. Information for pure or complicated HSPs were available in 440 patients, including 378 (85.90%) pure patients and 62 (14.10%) complicated patients. Our re-analysis suggests that most *ATL1* gene mutations usually display a pure phenotype, but *ATL1* gene mutation can also been found in patients with complicated phenotype of HSPs. The complicated symptoms of HSPs-SPG3A patients included seizure, optic atrophy, sensory impairment, mental retardation, ataxia, distal atrophy and peripheral axonal neuropathy (Additional file 1: Table S1). Additionally, we found that distal atrophy is the most common symptom in complicated HSPs-SPG3A patients (69.35%, 43/62).

The early-onset and relatively non-progressive nature of lower extremity spasticity in HSPs-SPG3A patients closely resembles symptoms of patients with spastic diplegic cerebral palsy. Because of this, many HSPs-SPG3A cases have been misdiagnosed as cerebral palsy even when there is no antecedent of a perinatal sentinel event and no lesions detected on brain imaging [10, 28, 31, 47]. However, reaching a HSP diagnosis in paediatric cases is challenging, especially in the absence of a positive family history. However, the occurrence of a de novo *ALT1* gene mutation must be considered in patients with spastic diplegic cerebral palsy, when other causes can not be identified.

Disease severity in HSPs-SPG3A patients is most commonly mild, although the severity of spasticity increases with disease duration. In general, the onset of disease symptoms in children has a long phase of relatively slow progression. In many cases, symptoms remain unchanged up to old age. Additionally, there are some asymptomic cases that contain the *ATL1* gene mutations. Incomplete penetrance has been previously reported in 10.56% HSPs families [8, 12, 15, 24, 27, 45]. The scarce penetrance of the mutations favours a modulator gene or strong epigenetic factor hypothesis, which may influence the phenotype. However, previous reports have shown the existence of some severe symptoms in HSPs-SPG3A patient. For example, Haberlova et al. reported a HSPs-SPG3A patient with a severe and early complicated phenotype, which was caused by the M408T mutation in *ATL1* gene [25]. Furthermore, a de novo G409D mutation in the *ATL1* gene exhibited an extremely severe spastic paraplegia combined with general hypertonia and hypokinesia since the neonatal period in one patient [47].

Linkage analysis suggests that mutations in the *ATL1* gene account for approximately 10% of ADHSP. The reported frequency of *ATL1* gene mutations varied from 2.9 to 38.7% though most studies reported a frequency of less than 15%. For example, we found that there were eight studies which reported a frequency of *ATL1* gene mutations less than 15%: 2.9% [27] in ADHSP families, 3.7% in ADSHP probands [45], 4.2% in HSP families [36], 8.3% in unrelated early onset pure ADHSP families [9], 6.6% in a heterogeneous population including both pure and complicated HSP phenotypes [21], 8.6% in ADHSP families [29], 11.3% in the ADHSP families [32], and 11.7% in ADHSP probands [44]. We also found four studies which reported a higher frequency of *ATL1* gene mutations: 20.0% in pure HSP [55], 20.0% (3/15) in early onset autosomal dominant HSP [13], 38.5% in SPG4-negative pure ADHSP families [11], and 38.7% in pure ADHSP families [15]. We also found that there was a difference in the frequency of *ATL1* gene mutations reported with different AAOs. For example, Namekawa et al. reported that the frequency of *ATL1* gene mutation in ADHSP was 6.6%, whereas the frequency was 13.5% in ADHSP families with onset before age 20, and it increased to 31.8% in ADHSP families with onset before age 10 [17]. The frequency variation may be caused by the differences in ethical criteria, number of patients and inclusion criteria, such as pure and complicated phenotypes of the patients, AAOs and *SPAST* mutations.

This study found that most *ATL1* gene mutations were located at exons 4, 7, 8 and 12, which is consistent with a previous study [29], which suggests that these exons should be given priority when performing molecular diagnosis. In addition, R239C (*n* = 31) [6, 9, 11, 13, 15, 17, 21, 22, 27–29, 31, 32, 36, 39, 45, 51, 53, 54, 56] and R495W (*n* = 14) [15–17, 21, 32, 36, 43, 50, 52, 54] mutations were the most commonly reported mutations in all studied families. Zhao et al. reported that the three families with R239C mutations were not apparently related and haplotype analysis did not exclude a distant founder effect [6]. Namekawa et al. reported that the R495W mutation could occur by independent mutational events [59]. Genetic testing should be performed in HSPs patients with very early-onset pure spastic paraplegia.

It is still not understood how the atlastin-1 protein functions and it is also unclear how autosomal dominant mutations in *ATL1* gene lead to the degeneration of upper motor neurons. In our analysis of the literature we found that all *ATL1* gene mutations except 3 (R217Q, c.35-3C < T, and exon 4 deletion) fell outside the GTPase-related motifs or the conserved motifs identified in the *ATL1* gene sequence which are thought to alter the structure of atlastin-1 and its interaction with other proteins [6, 11, 13, 16]. We find that most mutations are missense which suggests a gain-of-function pathogenic mechanism that is dependent on the position of the mutation, gene modifier and environmental factors [32]. This is supported by studies using yeast two-hybrid assay and co-immunoprecipitation of wild-type and p.del436N atlastin proteins which show that the p.del436N mutant protein can still oligomerize with wild-type atlastin, supporting a loss-of-function disease mechanism [23]. Atlastin-1 interacts with spastin (*SPG4*), suggesting that they may be a part of a common biological cascade whose disruption can result in motor neuron death [60].

## Conclusions

Our reanalysis demonstrates that most HSPs-SPG3A patients exhibited a pure autosomal dominant HSP with early AAO. The causal *ATL1* gene mutations are missense mutations and exons 4, 7, 8 and 12 should be prioritized for genetic testing. We find that there is no clear genotype-phenotype correlation.

## Additional file

Additional file 1: Table S1. Correlation between clinical and genetic characteristics in patients with mutant *ATL1*. (DOC 562 kb)

## Abbreviations

AAO: Age at onset
AD: Autosomal dominant
ADHSP: Autosomal dominant HSP
AR: Autosomal recessive
GTPases: Guanosine triphosphatases
HSP: Hereditary spastic paraplegias
SPG3A: Spastic paraplegia 3A
