# Supplementary material for: Clinical features and genotype-phenotype correlation analysis in patients with ATL1 mutations: A literature reanalysis
Source: Transl Neurodegener. 2017 Apr 4;6:9. doi: 10.1186/s40035-017-0079-3 (PMC5379717; doi:10.1186/s40035-017-0079-3)
Supplement: Additional file 1: Table S1. — Correlation between clinical and genetic characteristics in patients with mutant ATL1. (DOC 562 kb) [file 40035_2017_79_MOESM1_ESM.doc]

**Table S1: Correlation between clinical and genetic characteristics in patients with mutant *ATL1***

| Mutation | Mode | Code | Exon | Family | Patient | AAO | Age/Gender | P/C# | UL | LL | Babinski sign | Urinary urgency | Other | Reference |
| --- | --- | --- | --- | --- | --- | --- | --- | --- | --- | --- | --- | --- | --- | --- |
| G353A | AR | R118Q | 3 | 1 | 1 | 1 | 45/M | P | - | + | + | + | - | 48 |
|  |  |  |  |  | 2 | 1 | 31/M | P | - | + | + | + | - |  |
|  |  |  |  |  | 3 | 1 | 43/M | P | - | + | + | + | - |  |
|  |  |  |  |  | 4 | 1 | 12/M | P | - | + | + | - | - |  |
|  |  |  |  |  | 5 | 1 | 10/M | P | - | + | + | - | - |  |
|  |  |  |  |  | 6 | 1 | 3/M | P | - | + | + | - | - |  |
| T452C | AD | F151S | 4 | W | n=11 | 1-33 | NA/NA | P | - | + | + | 3/11 | - | 13 |
| T452C | AD | F151S | 4 | 1 | 1 | 4 | 49/NA | C | - | + | + | - | Pes Cavus/ Distal atrophy | 21 |
| G458C | AD | S153T | 4 | F4 | 1 | 4 | NA/NA | P | NA | NA | NA | NA | NA | 54 |
|  |  |  |  |  | 2 | NA | NA/NA | P | NA | NA | NA | NA | NA |  |
|  |  |  |  |  | 3 | NA | NA/NA | P | NA | NA | NA | NA | NA |  |
| C460G | AD | Q154E | 4 | 1 | 1 | 1 | NA/M | P | NA | NA | NA | NA | NA | 33 |
|  |  |  |  |  | 2 | 2 | NA/M | P | NA | NA | NA | NA | NA |  |
| C467T | AD | T156I | 4 | 1 | n=6 | Infancy | 5-61/NA | P | - | + | + | - | Pes Cavus | 14 |
| T470G | AD | L157W | 4 | 1 | 1 | Childhood | 34/F | P | - | + | + | - | - | 18 |
|  |  |  |  |  | 2 | 10month | 8/M | P | - | + | + | - | - |  |
| T470G | AD | L157W | 4 | 1 | 1 | Infancy | 41/NA | C | + | + | + | - | Distal atrophy | 21 |
|  |  |  |  |  | 2 | 1 | 3/NA | P | NA | + | + | - | - |  |
| G473C | AD | R158T | 4 | F31 | 1 | 2 | NA/NA | P | NA | NA | NA | NA | NA | 54 |
|  |  |  |  |  | 2 | NA | NA/NA | P | NA | NA | NA | NA | NA |  |
| G481C | AD | A161P | 4 | 1 | n=4 | Childhood | NA/NA | P | - | + | + | - | - | 13 |
| G481C | AD | A161P | 4 | 2 | n=3 | 45-55 | NA/NA | P | 1/3 | + | + | 1/3 | - | 13 |
| G481C | AD | A161P | 4 | 1 | II:3 | Childhood | 38/F | P | - | + | + | - | - | 11 |
|  |  |  |  |  | I:2 | NA | NA/F | P | - | + | + | NA | - |  |
|  |  |  |  |  | III:1 | NA | NA/M | P | - | + | + | NA | Supinated feet, learning difficulty |  |
|  |  |  |  |  | II:1 | NA | NA/F | P | - | + | NA | - | learning difficulty |  |
| G481C | AD | A161P | 4 | 2 | 1 | 55 | NA/F | P | - | + | + | + | - | 11 |
|  |  |  |  |  | 2 | 45 | NA/M | NA | NA | NA | NA | NA | NA |  |
|  |  |  |  |  | 3 | NA | NA/M | NA | NA | NA | NA | NA | NA |  |
| A484C | AD | T162P | 4 | 1 | 1 | Infancy | NA/NA | P | NA | NA | NA | NA | NA | 17 |
| T488C | AD | V163A | 4 | 1 | 1 | 1.5 | 30/M | P | - | + | + | - | hyperlordosis | 41 |
|  |  |  |  |  | 2 | 7 | 54/F | P | - | + | + | - | Pes Cavus |  |
| G493A | AD | A165T | 4 | 1 | 1 | Infancy | 34/M | P | - | + | NA | + | NA | 29 |
|  |  |  |  |  | 2 | NA | NA/M | P | - | NA | NA | + | NA |  |
| C565G | AD | H189D | 5 | 1 | n=8 | 1-6 | NA/NA | P | - | + | + | - | Flat feet | 22 |
| A572G | AD | Q191R | 5 | 1 | 1 | Infancy | 39/NA | C | - | + | + | - | Distal atrophy | 21 |
| A587G | AD | Y196C | 6 | 1 | 1 | 35 | NA/NA | C | NA | NA | NA | NA | Seizure, MRI white matter | 36 |
| G650A | AD | R217Q | 7 | 1 | NA | 8.3*(n=11) | NA/NA | P | NA | NA | NA | NA | - | 7 |
| G650A | AD | R217Q | 7 | 1 | n=11 | 3-29(n=11) | NA/NA | P | - | + | + | - | - | 13 |
| C715T | AD | R239C | 7 | S | NA | <10(n=8) | NA/NA | P | NA | NA | NA | NA | - | 6 |
| C715T | AD | R239C | 7 | A | NA | <10(n=7) | NA/NA | P | NA | NA | NA | NA | - | 6 |
| C715T | AD | R239C | 7 | M | NA | <10(n=3) | NA/NA | P | NA | NA | NA | NA | - | 6 |
| C715T | AD | R239C | 7 | 1 | I:2 | 3 | 62/F | P | - | + | - | - | - | 9 |
|  |  |  |  |  | II:1 | 3 | 40/M | P | - | + | + | - | - |  |
|  |  |  |  |  | II:2 | 3 | 35/M | P | - | + | + | - | - |  |
|  |  |  |  |  | II:3 | 3 | 33/F | P | - | + | + | - | - |  |
|  |  |  |  |  | III:1 | 3 | 14/F | P | - | + | + | - | - |  |
|  |  |  |  |  | III:1 | 3 | 11/M | P | - | + | + | - | - |  |
| C715T | AD | R239C | 7 | 1 | 1 | 1 | 8/M | P | - | + | + | NA | - | 11 |
|  |  |  |  |  | 2 | 3-4 | NA/M | P | - | + | + | NA | - |  |
|  |  |  |  |  | 3 | Infancy | NA/M | NA | NA | NA | NA | NA | NA |  |
| C715T | AD | R239C | 7 | 2 | 1 | Childhood | 53/M | P | - | + | + | + | - | 11 |
|  |  |  |  |  | 2 | 3 | NA/M | P | - | + | + | NA | Supinated feet |  |
| C715T | AD | R239C | 7 | C | n=22 | 2-50 | NA/NA | P | - | + | + | - | - | 13 |
| C715T | AD | R239C | 7 | 1 | n=6 | 3 | NA/NA | P | - | + | + | - | - | 13 |
| C715T | AD | R239C | 7 | 1 | 1 | 1 | NA/NA | P | - | + | NA | NA | NA | 15 |
|  |  |  |  |  | 2 | 3 | NA/NA | P | - | + | NA | NA | NA |  |
|  |  |  |  |  | 3 | 4 | NA/NA | P | - | + | NA | NA | NA |  |
| C715T | AD | R239C | 7 | 1 | 2 | <7 | NA/NA | P | NA | NA | NA | NA | NA | 17 |
| C715T | AD | R239C | 7 | 1 | 1 | Infancy | 61/NA | P | - | + | + | - | - | 21 |
|  |  |  |  |  | 2 | Infancy | 36/NA | P | - | + | + | - | - |  |
|  |  |  |  |  | 3 | Infancy | 33/NA | P | - | + | + | - | - |  |
|  |  |  |  |  | 4 | 1 | 8.5/NA | P | - | + | + | - | - |  |
|  |  |  |  |  | 5 | 1 | 4.5/NA | P | - | + | + | - | - |  |
| C715T | AD | R239C | 7 | 2 | n=3 | 1-6 | NA/NA | P | - | + | + | - | Flat feet | 23 |
| C715T | S | R239C | 7 | 1 | 1 | 1 | NA/NA | P | NA | NA | NA | NA | NA | 27 |
| C715T | AD | R239C | 7 | 1 | 1 | Childhood | 40/M | P | - | + | + | - | - | 28 |
|  |  |  |  |  | 2 | 3-4 | 6/M | P | - | + | + | - | - |  |
|  |  |  |  |  | 3 | 3 | 9/F | P | - | + | + | - | - |  |
| C715T | AD | R239C | 7 | 1 | 1 | Infancy | 71/M | P | - | + | NA | + | Pes cavus | 29 |
| C715T | AD | R239C | 7 | 1 | 1 | Infancy | 28/F | P | - | + | + | - | - | 31 |
|  |  |  |  |  | 2 | Childhood | 50/F | P | NA | + | + | NA | - |  |
|  |  |  |  |  | 3 | 2 | 38/M | P | - | + | + | NA | - |  |
| C715T | AD | R239C | 7 | 1 | 1 | 8 | NA/NA | NA | NA | NA | NA | NA | NA | 32 |
| C715T | AD | R239C | 7 | 1 | 1 | 4 | NA/NA | NA | NA | NA | NA | NA | NA | 32 |
| C715T | AD | R239C | 7 | 1 | 1 | 3 | NA/NA | NA | NA | NA | NA | NA | NA | 32 |
| C715T | AD | R239C | 7 | 1 | 1 | 5 | NA/NA | NA | NA | NA | NA | NA | NA | 32 |
| C715T | AD | R239C | 7 | 1 | 1 | 10 | NA/NA | P | NA | NA | NA | NA | NA | 36 |
| C715T | AD | R239C | 7 | 2 | 1 | 15 | NA/NA | P | NA | NA | NA | NA | NA | 36 |
| C715T | AD | R239C | 7 | 1 | 1 | 6 | 56/F | P | - | + | + | - | Cerebral hypometabolsim | 40 |
|  |  |  |  |  | 2 | 6 | 54/M | P | - | + | + | - | Cerebral hypometabolsim |  |
| C715T | AD | R239C | 7 | 1 | NA | 1 | NA/NA | NA | NA | NA | NA | NA | NA | 43 |
| C715T | S | R239C | 7 | 1 | NA | <10 | NA/NA | P | NA | NA | NA | NA | NA | 45 |
| C715T | AD | R239C | 7 | 1 | 1 | infancy | 50/M | P | - | + | + | + | - | 51 |
|  |  |  |  |  | 2 | 3 | 17/F | P | - | + | + | - | - |  |
| C715T | AD | R239C | 7 | 1 | 1 | 2 | 2.2/F | P | - | NA | NA | - | - | 55 |
| C715T | AD | R239C | 7 | 1 | 1 | 4 | 56/M | P | + | + | + | NA | - | 53 |
|  |  |  |  |  | 2 | 3 | 54/F | P | - | + | + | NA | - |  |
|  |  |  |  |  | 3 | 6 | 52/F | P | - | + | + | NA | - |  |
|  |  |  |  |  | 4 | 1 | 49/F | P | - | + | + | NA | - |  |
|  |  |  |  |  | 5 | 2 | 30/M | P | - | + | + | NA | - |  |
|  |  |  |  |  | 6 | 5 | 26/F | P | - | + | - | NA | - |  |
|  |  |  |  |  | 7 | 4 | 21/M | P | - | + | - | NA | - |  |
|  |  |  |  |  | 8 | 5 | 23/M | P | - | + | + | NA | - |  |
|  |  |  |  |  | 9 | 4 | 18/M | P | - | + | + | NA | - |  |
|  |  |  |  |  | 10 | 1 | 6/M | P | - | + | - | NA | - |  |
| C715T | AD | R239C | 7 | F9 | 1 | 3 | NA/NA | P | NA | NA | NA | NA | NA | 54 |
|  |  |  |  |  | 2 | NA | NA/NA | P | NA | NA | NA | NA | NA |  |
| C715T | AD | R239C | 7 | F11 | 1 | 3 | NA/NA | P | NA | NA | NA | NA | NA | 54 |
|  |  |  |  |  | 2 | NA | NA/NA | P | NA | NA | NA | NA | NA |  |
|  |  |  |  |  | 3 | NA | NA/NA | P | NA | NA | NA | NA | NA |  |
|  |  |  |  |  | 4 | NA | NA/NA | P | NA | NA | NA | NA | NA |  |
| C715T | AD | R239C | 7 | 1 | 1 | 1.5 | NA/NA | P | NA | NA | NA | NA | NA | 56 |
| G716T | AD | R239L | 7 | 1 | 1 | 1.5 | 55/M | P | + | + | NA | + | - | 29 |
|  |  |  |  |  | 2 | NA | NA/M | NA | - | NA | NA | NA | NA |  |
| G716T | AD | R239L | 7 | 2 | 1 | Infancy | 9/M | P | - | + | NA | + | NA | 29 |
|  |  |  |  |  | 2 | Infancy | NA/F | C | - | NA | NA | NA | Distal dystrophy, optic atrophy |  |
| A740C | AD | H247P | 8 | 1 | n=2 | 3 | NA/NA | P | - | + | + | 1/2 | - | 13 |
| A740C | AD | H247P | 8 | 1 | II:2 | 3 | 37/F | P | - | + | + | - | Supinated | 11 |
|  |  |  |  |  | III:1 | 15 | 5/F | P | - | + | + | NA | Supinated |  |
| A740G | AD | H247R | 8 | 1 | 1 | NA | NA/NA | P | - | + | NA | NA | NA | 29 |
| A740G | AD | H247R | 8 | F65 | 1 | 10 | NA/NA | C | NA | NA | NA | NA | NA | 54 |
|  |  |  |  |  | 2 | NA | NA/NA | NA | NA | NA | NA | NA | NA |  |
|  |  |  |  |  | 3 | NA | NANA | NA | NA | NA | NA | NA | NA |  |
| T749C | AD | L250P | 8 | 1 | 1 | 6 | 69/NA | P | - | + | + | - | - | 21 |
|  |  |  |  |  | 2 | 5 | 37/NA | P | - | + | + | - | - |  |
|  |  |  |  |  | 3 | 5 | 39/NA | P | - | + | + | - | - |  |
|  |  |  |  |  | 4 | 2 | 15/NA | P | + | + | + | - | - |  |
|  |  |  |  |  | 5 | 6 | 12/NA | P | - | + | + | - | - |  |
|  |  |  |  |  | 6 | 5 | 10/NA | P | - | + | + | - | - |  |
| C751A | AD | Q251K | 8 | 1 | 1 | Asymptomic | - | - | - | - | - | - | Incomplete | 15 |
|  |  |  |  |  | 2 | 1 | NA/NA | P | - | + | NA | NA | NA |  |
|  |  |  |  |  | 3 | 5 | NA/NA | P | - | + | NA | NA | NA |  |
|  |  |  |  |  | 4 | 6 | NA/NA | P | - | + | NA | NA | NA |  |
|  |  |  |  |  | 5 | 10 | NA/NA | P | - | + | NA | NA | NA |  |
|  |  |  |  |  | 6 | 14 | NA/NA | P | - | + | NA | NA | NA |  |
| G757A | AD | V253I | 8 | 1 | 1 | Asymptomic | NA/NA | - | - | - | NA | NA | Incomplete | 15 |
| G757A | AD | V253I | 8 | 1 | 1 | 5 | NA/NA | P | NA | NA | NA | NA | NA | 17 |
|  |  |  |  |  |  | 6 | NA/NA | P | NA | NA | NA | NA | NA |  |
| G757A | AD | V253I | 8 | 1 | 1 | 25 | 41/M | P | - | + | + | NA | - | 24 |
|  |  |  |  |  | 2 | 6 | 12/M | P | - | + | + | NA | - |  |
|  |  |  |  |  | 3 | Asympotomic | 72/M | - | - | - | - | - | Incomplete |  |
| G757A | AD | V253I | 8 | 1 | 1 | 10/NA | NA/NA | P | NA | NA | NA | NA | NA | 32 |
| G757A | AD | V253I | 8 | 1 | 1 | 36 | NA/NA | C | NA | NA | NA | NA | ataxia | 46 |
| G757A | AD | V253I | 8 | 1 | 1 | NA | NA/F | NA | NA | NA | NA | NA | NA | 49 |
|  |  |  |  |  | 2 | 0.5 | NA/M | C | NA | NA | NA | NA | myopathy |  |
|  |  |  |  |  | 3 | 40 | NA/M | P | NA | NA | NA | NA | NA |  |
|  |  |  |  |  | 4 | NA | NA/M | NA | NA | NA | NA | NA | NA |  |
|  |  |  |  |  | 5 | - | 55/F | - | - | - | - | - | Incomplete |  |
|  |  |  |  |  | 6 | - | NA/F | - | - | - | - | - | Incomplete |  |
|  |  |  |  |  | 7 | NA | NA/M | NA | NA | NA | NA | NA | - |  |
|  |  |  |  |  | 8 | NA | NA/F | NA | NA | NA | NA | NA | - |  |
|  |  |  |  |  | 9 | - | NA/F | - | - | - | - | - | Incomplete |  |
|  |  |  |  |  | 10 | NA | NA/M | NA | NA | NA | NA | NA | - |  |
|  |  |  |  |  | 11 | NA | NA/F | NA | NA | NA | NA | NA | - |  |
|  |  |  |  |  | 12 | 11 | NA/M | NA | NA | NA | NA | NA | - |  |
|  |  |  |  |  | 13 | NA | NA/F | NA | NA | NA | NA | NA | - |  |
| G757A | AD | V253I | 8 | 1 | 1 | 20s | NA/M | P | - | + | + | + | - | 52 |
| A773G | AD | H258R | 8 | T | NA | 1-6(n=17) | NA/NA | P | NA | NA | NA | NA | - | 6 |
| T773G | AD | H258R | 8 | 1 | 1 | 1.5 | 49/NA | C | - | + | + | - | Distal atrophy | 21 |
|  |  |  |  |  | 2 | 1.5 | 25/NA | C | - | + | + | - | Distal atrophy |  |
|  |  |  |  |  | 3 | 1 | 22/NA | C | - | + | + | - | Distal atrophy |  |
| A773G | AD | H258R | 8 | 1 | NA | <10 | NA/NA | P | NA | NA | NA | NA | NA | 45 |
| C777A | AD | S259Y | 8 | P | NA | <3(n=13) | NA/NA | P | NA | + | + | NA | - | 6 |
| C776T | AD | S259Y | 8 | 1 | 1 | Infancy | NA/NA | P | - | + | NA | NA | NA | 15 |
|  |  |  |  |  | 2 | 2 | NA/NA | P | - | + | NA | NA | NA |  |
|  |  |  |  |  | 3 | 6 | NA/NA | NA | - | + | NA | NA | NA |  |
|  |  |  |  |  | 2 | 2 | NA/NA | P | - | + | NA | NA | NA |  |
| T776G | AD | S259F | 8 | 1 | 1 | 25 | 73/F | P | - | + | NA | - | - | 29 |
| T944G | AD | I315S | 9 | R | 1 | <9 | NA/NA | P | - | + | + | - | - | 13 |
| T944G | AD | I315S | 9 | 1 | 1 | Childhood | NA/NA | P | NA | NA | NA | NA | NA | 17 |
| C1006T | AD | Y336H | 10 | 1 | 1 | 1 | 63/NA | C | + | + | - | - | Distal atrophy | 21 |
|  |  |  |  |  | 2 | 1 | 34/NA | C | - | + | + | - | Distal atrophy |  |
| C1006T | AD | Y336H | 10 | 1 | 1 | 2 | NA/NA | NA | NA | NA | NA | NA | NA | 43 |
| C1006T | AD | Y336H | 10 | 1 | 1 | 1 | NA/NA | NA | NA | NA | NA | NA | NA | 43 |
| C1025A | AD | P342Q | 10 | 1 | 1 | 3 | 56/M | P | - | + | + | + | Pes planus, Hyperlordosis Restless syndrome | 41 |
|  |  |  |  |  | 2 | 1.5 | 14/M | P | - | + | + | - | Pes planus, restless syndrome |  |
| C1030T | S | P344S | 10 | 1 | 1 | 1 | 17/F | C | + | + | + | + | axonal neuropathy, cognitive impairment, pes cavus, distal amyotrophy | 38 |
| T1036G | AD | S346A | 10 | 1 | 1 | 0 | 55/F | P | - | + | + | NA | Pes cavus | 53 |
|  |  |  |  |  | 2 | 0 | 35/F | P | - | + | + | NA | Pes cavus |  |
|  |  |  |  |  | 3 | 0 | 33/F | P | - | + | + | NA | Pes cavus |  |
|  |  |  |  |  | 4 | 0 | 31/F | P | - | + | + | NA | Pes cavus |  |
|  |  |  |  |  | 5 | 0 | 12/F | P | - | + | + | NA | Pes cavus |  |
|  |  |  |  |  | 6 | 0 | 5/F | P | - | + | + | NA | Pes cavus |  |
| T1040C | AD | M347T | 10 | 1 | 1 | 11 | 47/F | C | - | + | + | - | Axonal neuropathy, Sensory impairment | 35 |
|  |  |  |  |  | 2 | Childhood | NA/M | C | 0 | NA | NA | - | Axonal neuropathy |  |
|  |  |  |  |  | 3 | 11 | 53/F | C | - | + | + | - | Axonal neuropathy, Sensory impairment |  |
|  |  |  |  |  | 4 | 11 | 12/F | P | - | + | + | - | - |  |
|  |  |  |  |  | 5 | 2 | 10/M | P | - | + | + | - | - |  |
|  |  |  |  |  | 6 | 25 | 25/F | C | - | + | - | - | Axon neuropathy |  |
| T1040C | S | M347T | 10 | 1 | 1 | infancy | 15/M | C | - | + | NA | - | Neuropathy | 51 |
| C1065A | S | N355K | 11 | 1 | 1 | 1.2 | 2.1/M | P | - | NA | NA | - | - | 55 |
| C1065A | S | N355K | 11 | 1 | 1 | 2 | 3.5/M | P | - | NA | NA | - | - | 55 |
| T1123C | AD | C375R | 12 | 1 | 1 | 1.5 | NA/NA | P | NA | NA | NA | NA | NA | 17 |
| C1193A | AD | S398Y | 12 | 014 | n=11 | 1.3-50 | NA/NA | P | 3/11 | + | + | 5/11 | - | 13 |
| C1193A | AD | S398Y | 12 | 1 | 1 | 1 | NA/NA | P | - | + | NA | NA | NA | 15 |
| C1193T | S | S398F | 12 | 1 | 1 | 0.5 | 4/F | C | - | + | + | - | Distal atrophy | 30 |
| A1220G | AD | K407R | 12 | 1 | 1 | 2-10(n=6) | NA/M(n=1), F(n=5) | P | NA | NA | NA | NA | Incomplete | 27 |
|  |  |  |  |  | 1 | Asymptomic | - | - | NA | NA | NA | NA | NA |  |
| A1222G | AD | M408V | 12 | 1 | II:3 | Infancy | 39/F | C | + | + | + | - | Distal atrophy of four limbs | 10 |
|  |  |  |  |  | III:2 | Infancy | 16/M | C | + | + | - | - | Distal atrophy of four limbs |  |
|  |  |  |  |  | III:3 | Infancy | 15/F | P | - | + | + | - | - |  |
|  |  |  |  |  | III:4 | Infancy | 6/M | P | - | + | + | - | - |  |
|  |  |  |  |  | III:5 | Infancy | 3/F | P | - | + | + | - | - |  |
|  |  |  |  |  | III:6 | Infancy | 1.25/M | P | - | + | + | - | - |  |
| A1222G | AD | M408V | 12 | 1 | n=6 | walkingdelay | NA/NA | C | LMN | + | + | - | Distal atrophy | 13 |
| T1223C | AD | M408T | 12 | 1 | 1 | 0.5 | 4.5/NA | C | + | + | - | - | Distal atrophy | 21 |
| A1222G | AD | M408T | 12 | 1 | 1 | 3m | 7/M | C | + | + | + | - | Distal atrophy | 25 |
|  |  |  |  |  | 2 | NA | NA/M | P | NA | NA | NA | NA | NA |  |
| G1226A | S | G409D | 12 | 1 | 1 | infancy | 12/M | C | + | + | + | - | Severe, atrophy in all limbs | 47 |
| G1228A | AD | G410R | 12 | 1 | I:7 | 15 | 84/F | NA | NA | NA | NA | NA | NA | 19 |
|  |  |  |  |  | II:2 | 3 | 57/M | C | - | + | - | NA | Muscular atrophy |  |
|  |  |  |  |  | II:3 | 3 | 59/M | NA | NA | NA | NA | NA | NA |  |
|  |  |  |  |  | II:5 | 4 | 35/F | NA | NA | NA | NA | NA | NA |  |
|  |  |  |  |  | II:9 | 12 | 43/M | C | - | + | - | NA | Muscular atrophy |  |
|  |  |  |  |  | III:2 | 11 | 25/F | C | - | + | + | NA | Muscular atrophy |  |
|  |  |  |  |  | III:7 | 7 | 24/M | C | - | + | + | NA | Muscular atrophy |  |
|  |  |  |  |  | III:8 | 1-2 | 20/F | C | NA | + | - | NA | Muscular atrophy |  |
|  |  |  |  |  | IV:1 | 1-2 | 6/M | C | - | + | + | NA | Muscular atrophy |  |
| A1237C | AD | F413V | 12 | 1 | 1 | 6 | NA/NA | C | NA | NA | NA | NA | Neuropathy | 32 |
|  |  |  |  |  | 2 | NA | NA/NA | NA | NA | NA | NA | NA | - |  |
| T1239C | AD | F413L | 12 | 2 | 1 | Infancy | NA/NA | P | - | + | NA | NA | NA | 15 |
|  |  |  |  |  | 2 | Infancy | NA/NA | P | - | + | NA | NA | NA |  |
| C1242G | AD | S414R | 12 | 1 | 1 | NA | NA/NA | NA | NA | NA | NA | NA | NA | 44 |
| C1243T | AD | R415W | 12 | 1 | 1 | 16 | NA/NA | NA | NA | NA | NA | NA | NA | 32 |
| C1243T | AD | R415W | 12 | 1 | IV:10 | 3 | 22/F | P | - | + | + | NA | Incomplete | 12 |
|  |  |  |  |  | IV:9 | 4 | 32/M | P | NA | + | NA | NA | - |  |
|  |  |  |  |  | III:9 | 5 | 49/M | P | NA | + | NA | NA | - |  |
|  |  |  |  |  | III:4 | Infancy | 63/M | P | NA | NA | NA | NA | NA |  |
|  |  |  |  |  | II:1 | Infancy | 75/M | P | NA | NA | NA | NA | NA |  |
|  |  |  |  |  | II:6 | Asymptomic | - | - | - | - | - | - | - |  |
|  |  |  |  |  | III:1 | Asymptomic | - | - | - | - | - | - | - |  |
|  |  |  |  |  | III:2 | Asymptomic | - | - | - | - | - | - | - |  |
|  |  |  |  |  | III:8 | Asymptomic | - | - | - | - | - | - | - |  |
|  |  |  |  |  | IV:1 | Asymptomic | - | - | - | - | - | - | - |  |
|  |  |  |  |  | IV:2 | Asymptomic | - | - | - | - | - | - | - |  |
|  |  |  |  |  | IV:3 | Asymptomic | - | - | - | - | - | - | - |  |
|  |  |  |  |  | IV:4 | Asymptomic | - | - | - | - | - | - | - |  |
| C1243T | AD | R415W | 12 | 1 | I:1 | Asymptomic | Deceased/M | - | NA | NA | NA | NA | NA | 42 |
|  |  |  |  |  | II:1 | Asymptomic | 43/M | - | - | - | - | NA | Incomplete |  |
|  |  |  |  |  | II:2 | 16 | 39/M | P | - | + | + | NA | - |  |
| C1243T | AD | R415W | 12 | 1 | 1 | 12 | NA/NA | P | NA | NA | NA | NA | NA | 50 |
| C1243T | AD | R415W | 12 | F13 | 1 | 12 | NA/NA | P | NA | NA | NA | NA | NA | 54 |
|  |  |  |  |  | 2 | NA | NA/NA | P | NA | NA | NA | NA | NA |  |
|  |  |  |  |  | 3 | NA | NA/NA | P | NA | NA | NA | NA | NA |  |
| C1243T | AD | R415W | 12 | F117 | 1 | 3 | NA/NA | C | NA | NA | NA | NA | Axon neuropathy | 54 |
|  |  |  |  |  | 2 | NA | NA/NA | NA | NA | NA | NA | NA | NA |  |
|  |  |  |  |  | 3 | NA | NA/NA | NA | NA | NA | NA | NA | NA |  |
| A1244 | AD | R415Q | 12 | 1 | II:2 | Asymptomic | 54/M | - | - | - | - | NA | Incomplete | 42 |
|  |  |  |  |  | II:3 | Asymptomic | 42/F | - | - | - | + | NA | - |  |
|  |  |  |  |  | III:1 | Asymptomic | 28/F | - | - | - | - | NA | - |  |
|  |  |  |  |  | III:2 | Asymptomic | 22/F | - | - | - | - | NA | - |  |
|  |  |  |  |  | III:4 | 5 | 21/F | P | + | + | + | NA | - |  |
|  |  |  |  |  | III:5 | 10 | 16/F | P | - | + | + | NA | - |  |
|  |  |  |  |  | III:7 | 5 | 13/F | P | + | + | + | NA | - |  |
|  |  |  |  |  | III:8 | Asymptomic | 10/M | - | - | - | - | NA | - |  |
|  |  |  |  |  | III:9 | 5 | 25/M | P | - | + | + | NA | - |  |
| C1246T | AD | R416C | 12 | 1 | 1 | 45 | 53/M | C | - | + | NA | - | Mental retardation, TCC¶ | 34 |
|  |  |  |  |  | 2 | 44 | 66/M | C | - | + | NA | + | Mental retardation |  |
|  |  |  |  |  | 3 | 56 | 68/M | C | - | + | NA | + | Mental retardation |  |
|  |  |  |  |  | 4 | 51 | 56/F | C | - | + | NA | - | Mental retardation |  |
|  |  |  |  |  | 5 | 42 | 51/F | C | - | + | NA | - | Mental retardation |  |
|  |  |  |  |  | 6 | 38 | 56/M | C | - | + | NA | + | Mental retardation |  |
|  |  |  |  |  | 7 | 48 | 50/F | C | - | + | NA | - | Mental retardation |  |
|  |  |  |  |  | 8 | 46 | 51/M | C | - | + | NA | - | Mental retardation |  |
|  |  |  |  |  | 9 | 39 | 55/F | C | - | + | NA | - | Mental retardation |  |
|  |  |  |  |  | 10 | 41 | 49/M | C | - | + | NA | - | Mental retardation |  |
| C1246T | AD | R416C | 12 | 1 | 1 | Puberty | 52/F | P | NA | - | + | - | - | 37 |
|  |  |  |  |  | 2 | NA | NA/F | P | NA | NA | NA | NA | NA |  |
|  |  |  |  |  | 3 | NA | NA/M | P | NA | NA | NA | NA | NA |  |
| C1246T | AD | R416C | 12 | 1 | 1 | <10(n=10) | NA/NA | P | NA | NA | NA | NA | Incomplete | 45 |
|  |  |  |  |  |  | Late(n=3) | NA/NA | C | NA | NA | NA | NA | NA |  |
| G1247A | AD | R416H | 12 | 1 | 1 | 5 | 39/F | C | + | + | + | - | Cerebellar ataxia | 33 |
|  |  |  |  |  | 2 | 67 | 68/M | C | - | + | - | NA | ALS§ |  |
| T1308A | S | N436K | 12 | 1 | 1 | infancy | 3/M | P | - | + | + | - | - | 51 |
| A1319C | AD | N440T | 12 | 1 | 1 | 17 | NA/NA | P | NA | NA | NA | NA | NA | 32 |
| A1319C | AD | N440T | 12 | 3 | 1 | 6 | NA/NA | P | - | + | NA | NA | NA | 15 |
|  |  |  |  |  | 2 | 8 | NA/NA | P | - | + | NA | NA | NA |  |
|  |  |  |  |  | 3 | 8 | NA/NA | P | - | + | NA | NA | NA |  |
| A1376G | AD | Y459C | 12 | 1 | 1 | 3 | 29/F | P | - | + | + | - | - | 20 |
|  |  |  |  |  | 2 | Childhood | NA/M | NA | NA | NA | NA | NA | NA |  |
|  |  |  |  |  | 3 | NA | NA/M | NA | NA | NA | NA | NA | NA |  |
| G1406C | AD | G469A | 12 | 1 | 1 | NA | 63/NA | P | - | + | - | - | - | 21 |
|  |  |  |  |  | 2 | 2 | 40/NA | C | + | + | - | - | Distal atrophy |  |
|  |  |  |  |  | 3 | 2 | 29/NA | C | + | + | - | - | Distal atrophy |  |
|  |  |  |  |  | 4 | 2 | 10/NA | P | - | + | + | - | - |  |
| G1445T | AD | G482V | 12 | 1 | 1 | Asymptomic | 63/NA | - | - | + | + | - | Incomplete | 21 |
|  |  |  |  |  | 2 | 9 | 61/NA | P | + | + | + | - | - |  |
|  |  |  |  |  | 3 | 1.5 | 32/NA | C | - | + | + | - | Epilepsy |  |
|  |  |  |  |  | 4 | 3 | 34/NA | P | - | + | + | - | - |  |
|  |  |  |  |  | 5 | 1 | 12/NA | P | - | + | + | - | - |  |
| C1483T | AD | R495W | 12 | 1 | 1 | Infancy | NA/NA | P | - | + | NA | NA | Incomplete | 15 |
|  |  |  |  |  | 2 | 4 | NA/NA | P | - | + | NA | NA | NA |  |
|  |  |  |  |  | 3 | 5 | NA/NA | P | - | + | NA | NA | NA |  |
|  |  |  |  |  | 4 | 6 | NA/NA | P | - | + | NA | NA | NA |  |
|  |  |  |  |  | 5 | Asymptomic | 4/? | - | - | - | NA | NA | NA |  |
| C1483T | AD | R495W | 12 | 2 | 1 | Unkown | NA/NA | P | - | + | NA | NA | Incomplete | 15 |
|  |  |  |  |  | 2 | 6 | NA/NA | P | - | + | NA | NA | NA |  |
| C1483T | AD | R495W | 12 | 3 | 1 | 2 | NA/NA | P | - | + | NA | NA | Incomplete | 15 |
|  |  |  |  |  | 2 | 2 | NA/NA | P | - | + | NA | NA | NA |  |
|  |  |  |  |  | 3 | 10 | NA/NA | P | - | + | NA | NA | NA |  |
|  |  |  |  |  | 4 | 10 | NA/NA | P | - | + | NA | NA | NA |  |
|  |  |  |  |  | 5 | Asymptomic | 53/NA | - | - | - | NA | NA | NA |  |
| C1483T | AD | R495W | 12 | 4 | 1 | 2 | NA/NA | P | - | + | NA | NA | NA | 15 |
|  |  |  |  |  | 2 | Asymptomic | 50/NA | - | - | + | NA | NA | Incomplete |  |
| C1483T | AD | R495W | 12 | 1 | 1 | 3 | 14/M | P | - | + | + | - | scoliosis  Pes Planus | 16 |
|  |  |  |  |  | 2 | 7 | 12/F | P | - | + | + | - |  |
|  |  |  |  |  | 3 | 3 | 11/F | P | - | + | + | - |  |
| C1483T | AD | R495W | 12 | 1 | 1 | 7 | NA/NA | P | NA | NA | NA | NA | NA | 17 |
| C1483T | AD | R495W | 12 | 1 | 1 | 20 | 57/NA | C | - | + | + | - | Distal atrophy | 21 |
|  |  |  |  |  | 2 | 8 | 42/NA | C | - | + | + | - | Distal atrophy |  |
|  |  |  |  |  | 3 | 10 | 34/NA | C | - | + | + | - | Distal atrophy |  |
|  |  |  |  |  | 4 | 10 | 20/NA | C | - | + | - | - | Distal atrophy |  |
|  |  |  |  |  | 5 | NA | 12/NA | P | - | + | - | - | Distal atrophy |  |
| C1483T | AD | R495W | 12 | 1 | 1 | 5 | NA/NA | P | NA | NA | NA | NA | NA | 32 |
| C1483T | AD | R495W | 12 | 1 | 1 | 8 | NA/NA | P | NA | NA | NA | NA | NA | 36 |
| C1483T | AD | R495W | 12 | 1 | 1 | 9 | NA/NA | NA | NA | NA | NA | NA | NA | 43 |
| C1483T | S | R495W | 12 | 1 | 1 | 12 | NA/NA | NA | NA | NA | NA | NA | NA | 50 |
| C1483T | AD | R495W | 12 | 1 | 1 | 10’s | NA/F | P | - | + | NA | - | NA | 52 |
| C1483T | AD | R495W | 12 | F8 | 1 | 4 | NA/NA | P | NA | NA | NA | NA | NA | 54 |
|  |  |  |  |  | 2 | NA | NA/NA | P | NA | NA | NA | NA | NA |  |
|  |  |  |  |  | 3 | NA | NA/NA | P | NA | NA | NA | NA | NA |  |
|  |  |  |  |  | 4 | NA | NA/NA | P | NA | NA | NA | NA | NA |  |
|  |  |  |  |  | 5 | NA | NA/NA | P | NA | NA | NA | NA | NA |  |
|  |  |  |  |  | 6 | NA | NA/NA | P | NA | NA | NA | NA | NA |  |
|  |  |  |  |  | 7 | NA | NA/NA | P | NA | NA | NA | NA | NA |  |
|  |  |  |  |  | 8 | NA | NA/NA | P | NA | NA | NA | NA | NA |  |
| G1556A | AD | S519N | 13 | 1 | 1 | 10 | NA/NA | P | - | + | NA | NA | NA | 15 |
| 1306-1308delAAT | AD | N436del | 12 | 1 | 1 | 1-2 | 43/NA | P | + | + | + | - | - | 23 |
|  |  |  |  |  | 2 | 1-2 | 23/NA | P | - | + | + | - | - |  |
|  |  |  |  |  | 3 | 1-2 | 22/NA | P | - | + | + | - | - |  |
|  |  |  |  |  | 4 | 1-2 | 18/NA | P | - | + | + | - | - |  |
|  |  |  |  |  | 5 | 1-2 | 26/NA | P | - | + | + | - | - |  |
| 1306-1308delAAT | AD | N436del | 12 | 2 | 1 | 18 | 57/NA | P | - | + | + | - | - | 23 |
|  |  |  |  |  | 2 | 1.5 | 4/NA | P | + | + | + | - | - |  |
|  |  |  |  |  | 3 | NA | 36/NA | P | - | + | - | - | Pes Cavus |  |
|  |  |  |  |  | 4 | 16 | 63/NA | C | - | + | + | NA | Muscle atrophy |  |
| 1306-1308delAAT | AD | N436del | 12 | 1 | 1 | 1 | NA/NA | P | NA | NA | NA | NA | NA | 27 |
| Exon 4 del | NA | 140-174del | 4 | 1 | 1 | NA | NA/NA | NA | NA | NA | NA | NA | NA | 38 |
| c.35-3C>T | AD | G13fsX16 | Intron 2 | 1 | 1 | 44 | 45/M | P | - | + | + | + | - | 46/52 |
|  |  |  |  |  | 2 | Asymptomic | 17/F | - | - | - | - | - | Incomplete |  |
|  |  |  |  |  | 3 | Asymptomic | 15/M | - | - | - | - | - | Incomplete |  |
| 1466-1467insTG | AD | T490fsX508 | 12 | 1 | 1 | 5 | 17/F | NA | NA | NA | NA | NA | NA | 44 |
| 1474insG | AD | A492fsX522 | 12 | 1 | 1 | 1 | 36/NA | P | - | + | + | - | - | 21 |
| 1504-1505insG | AD | E502fs  X522 | 12 | 1 | 1 | 4 | 44/F | P | - | + | + | - | NA | 26 |
| 1504-1505insG | AD | E502fs  X522 | 12 | 1 | 1 | 2 | NA/NA | NA | NA | NA | NA | NA | NA | 43 |
| 1505_1506delAG | AD | E502fsX21 | 12 | 1 | 1 | 40 | 47/M | P | - | + | + | - | Restless legs syndrome | 41 |
|  |  |  |  |  | 2 | 13 | 57/F | P | + | + | + | + | Pes equines, hyperlordosis |  |
| 1520insA | AD | I507fsX522 | 12 | 1 | n=9 | 5-39 | NA/NA | P | 3/9 | + | + | - | - | 13 |
| 1520insA | AD | I507fsX522 | 12 | 1 | III:6 | 20 | 81/M | P | + | + | + | - | Incomplete | 8 |
|  |  |  |  |  | IV:2 | 5 | 65/M | P | - | + | + | + | - |  |
|  |  |  |  |  | IV:3 | Asymptomic | 62/F | - | - | + | - | - | - |  |
|  |  |  |  |  | IV:4 | 30 | 49/F | P | - | + | - | - | - |  |
|  |  |  |  |  | IV:5 | 10 | 52/M | P | + | + | + | + | - |  |
|  |  |  |  |  | IV:14 | 39 | 56/F | P | - | + | - | + | - |  |
|  |  |  |  |  | V:4 | 10 | 33/M | P | + | + | + | + | - |  |
|  |  |  |  |  | V:5 | 12 | 25/F | P | - | + | - | - | - |  |
|  |  |  |  |  | V:8 | Asymptomic | 25/F | - | - | + | - | - | - |  |

P/C#, pure/complicated form; * mean value; AAO, age at onset; UL, upper limbs; LL, lower limbs; NA, not available; AR, autosomal recessive; AD, autosomal dominant; S, Sporadic; ALS§, amyotrophic lateral sclerosis; TCC¶, thin corpus callosum
